# Exploring Bat–Virus Interactions: Insights from a Study in the Gobi Desert

**DOI:** 10.3390/pathogens14090870

**Published:** 2025-09-02

**Authors:** Sabrina Canziani, Davide Lelli, Paolo Agnelli, Claudio Augugliaro, Munkhtsetseg Bazarragchaa, Sandro Bertolino, Marco Carlomagno, Gantulga Davaakhuu, Massimo Delledonne, Fabrizio Gili, Renato Fani, Ana Moreno, Battogtokh Nasanbat, Francesco Riga, Marzia Rossato, Tiziana Trogu, Leonardo Vincenzi, Udval Uuganbayar, Antonio Lavazza, Marco Zaccaroni

**Affiliations:** 1 Istituto Zooprofilattico Sperimentale della Lombardia e dell’Emilia Romagna, Via Bianchi 9, 25124 Brescia, Italy; sabrina.canziani@izsler.it (S.C.); anamaria.morenomartin@izsler.it (A.M.); tiziana.trogu@izsler.it (T.T.); alavazza0@gmail.com (A.L.); 2 Department of Medicine and Surgery, University of Parma, Via Gramsci 14, 43126 Parma, Italy; 3 Natural History Museum, University of Florence, Via Romana 17, 50125 Florence, Italy; paoloagnelli55@gmail.com; 4 Department of Biology, Mongolian National University of Education, Ulaanbaatar 14191, Mongolia; claudiocites@gmail.com; 5 Department of Molecular Biology and Genetics, School of Bio-Medicine, Mongolian National University of Medical Sciences, Ulaanbaatar 14210, Mongolia; munkhtsetseg.ba@mnums.edu.mn (M.B.); udka1025@gmail.com (U.U.); 6 Department of Life Sciences and Systems Biology, University of Turin, Via Accademia Albertina 13, 10123 Turin, Italy; sandro.bertolino@unito.it (S.B.); fabrizio.gili@irsa.cnr.it (F.G.); 7 Department of Biotechnology, University of Verona, Strada le Grazie 15, 37134 Verona, Italy; marco.carlomagno@univr.it (M.C.); massimo.delledonne@univr.it (M.D.); marzia.rossato@univr.it (M.R.); leonardo.vincenzi@univr.it (L.V.); 8 Institute of Biology, Mongolian Academy of Sciences, Peace Avenue-54B, Bayanzurkh District, Ulaanbaatar 13330, Mongolia; gantulgad@mas.ac.mn (G.D.); battogtokhn@mas.ac.mn (B.N.); 9 Department of Biology, University of Florence, Via Madonna del Piano 6, 59100 Sesto Fiorentino, Italy; renato.fani@unifi.it (R.F.); marco.zaccaroni@unifi.it (M.Z.); 10 Italian Institute for Environmental Protection and Research (ISPRA), Via V. Brancati 48, 00144 Rome, Italy; francesco.riga@isprambiente.it

**Keywords:** bats, emerging viruses, Gobi Desert, MERS-related coronavirus, mammalian orthoreovirus, herpesvirus detection

## Abstract

In May 2022, an expedition was conducted in the Gobi Desert, Mongolia, to investigate the viral diversity of bats, recognized as reservoirs of emerging zoonotic viruses. Bats were captured in six oases using mist nets and were identified morphologically and molecularly. Fecal samples were collected and screened using molecular protocol targeting viral agents of relevance to human and animal health, including coronaviruses, orthoreoviruses, herpesviruses, adenoviruses, flaviviruses, phleboviruses, paramyxoviruses, pestiviruses, and Influenza A viruses. In total, 74 bats were sampled. The most represented bat genus was *Plecotus*, followed by *Hypsugo*, *Vespertilio*, and *Myotis*. Coronavirus RNA was detected in eleven samples (14.86%), *Mammalian orthoreovirus* RNA in two samples (2.70%), and herpesvirus DNA in three samples (4.05%). No other targeted viruses were detected. These data expand our understanding of viral circulation in bats from previously unstudied regions. By expanding our understanding of the viral diversity harbored by bats, this study contributes to ongoing efforts to better characterize their role in the ecology and evolution of emerging zoonotic viruses. Continuous surveillance in remote and biodiverse areas is essential to identify potential threats to public and animal health and to improve preparedness for future viral emergence.

## 1. Introduction

Bats belong to Chiroptera, among the largest orders in the class of mammals and second in number of species only to that of rodents. They are widespread on all continents except Antarctica [1] and are characterized by an extreme variety of species. In fact, more than 1450 species are recognized, which, according to the most recent phylogenetic studies, are divided into the two suborders Yinpterochiroptera (or Pteropodiformes) and Yangochiroptera (or Vespertilioniformes) [2].

The number of potential viruses in bats that can infect humans is comparable to the number of viruses also present in other species of mammals [3,4], and since each species is different, the viruses present in the different bat species are also not always the same. Furthermore, within the animal kingdom, there are several primate species among those identified as being susceptible to viruses closely related to those infecting humans, due to a high degree of homology in cellular membrane receptors involved in viral entry. As expected, based on phylogenetic relatedness, these comprise the lowland gorilla (*Gorilla gorilla gorilla*), the Sumatran orangutan (*Pongo abelii*), and the white-cheeked gibbon (*Nomascus leucogenys*).

Marine mammals such as the gray whale and the bottlenose dolphin are also at high risk; domestic animals such as cats, cattle, and sheep are at medium risk; while dogs, horses, and pigs are at low risk [5].

Despite this, bats are the most studied animals for the research of pathogenic viruses, mainly due to their peculiar physiological and ethological characteristics, which make them perfect reservoirs for their viruses, some of which could be transmitted to humans, although most likely indirectly.

The main reason for this interest in bats is due to the ease of intra- and interspecific transmission of viruses between populations, due to a complex phenomenon that depends on the host’s immune system and the intrinsic characteristics of viruses. Bats have, in fact, developed, over 53 million years, immune mechanisms that mean they have a benign relationship with the pathogens they host, allowing them to resist the disease [6]. This ability has been linked to their adaptation to flight [7]; the increase in metabolism during flight leads to an increase in free radicals in oxygen, making bats more prone to generating damaged DNA that suppresses the immune response [8]. Furthermore, bats are the only mammals capable of flying, and some of the species living in temperate zones are able to cover long distances during their seasonal migrations in search of the best summer and winter shelters. Different migration patterns within the same species allow the exchange of new viruses or variants between subpopulations of conspecific bats or other species [9]. Bat species living in temperate zones enter a state of deep torpor (hibernation) during the winter, and this characteristic allows the pathogens they host to overwinter, thus remaining persistently infected and able to further disseminate the viruses. The demographic structure of bats can also facilitate viral circulation within the bat population; in particular, the high population density and their gregarious behavior increase the possibility of intra- and interspecific transmission of viruses.

Bats shelter in cavities and crevices found in trees, caves, and artificial structures, such as mines, ruins, and even buildings more or less occupied by humans, both in rural areas and in historical–monumental buildings [10]. Due to their global distribution and ecological diversity, bats are frequently exposed to a wide spectrum of viral agents [11]; furthermore, since they are colonial animals, a large number of bats of the same and different species can be found inside a cave, and such close physical proximity facilitates the transmission and amplification of viruses [12]. Although bats are more frequent hosts of pathogens and can transmit them to other vertebrates and potentially also indirectly to humans [13], spillover events rarely occur since certain conditions must be met: (i) the reservoir host must be able to transmit the virus; (ii) the environmental conditions must be suitable for the survival of the pathogen; and (iii) the host exposed to the infection must be susceptible and come into contact with a sufficient amount of virus to trigger the infection [14]. The ancient origins of some zoonotic viruses present in bats, such as Lyssavirus [15], suggest a long history of coevolution.

Although many viruses are not directly transmitted from bats to humans or other animals, bats are considered natural reservoirs of viruses such as coronaviruses (including SARS-CoV and MERS-CoV), henipaviruses (Nipahvirus (NiV) and Hendravirus (HeV)), and filoviruses (Ebola and Marburg) [11] that can cause emerging infectious diseases and have a serious impact on human health.

Therefore, drawing from the experience gained through over a decade of monitoring bats in Northern Italy—an industrialized region with a high-density population marked by significant fragmentation and deterioration of the ecosystem and ecological niches—we planned to employ the same diagnostic methods to assess the diversity of viruses present in bat populations of the Gobi Desert, one of the most remote and least densely populated regions on the planet. Given the growing interest in bats and the complex dynamics inherent in their ability to carry and transmit various pathogens, an expedition was organized in 2022 to explore this vast and ecologically untouched area of Mongolia.

The mission aimed to investigate the wildlife and microbial diversity of the Great Gobi, a natural sanctuary that conserves many animal species. In this project, special attention was paid to the different bat species present in the area and to the possible detection of viral agents, especially of zoonotic interest, hosted by them. The primary aim of this study was to investigate the viral diversity in bat populations of the Gobi Desert, with a particular focus on identifying potential zoonotic viruses. This exploratory approach aims to broaden our understanding of bat-associated viruses in arid and sparsely populated regions, which remain largely unexplored in the scientific literature.

## 2. Materials and Methods

### 2.1. Sampling

**Figure 1:**
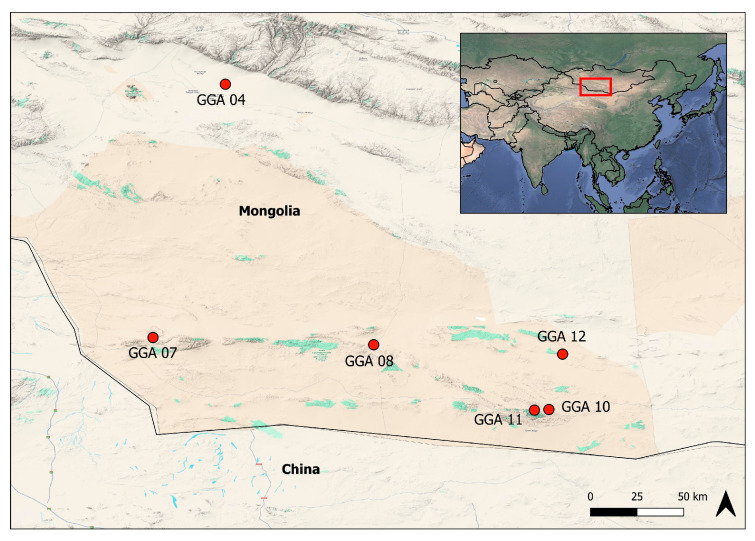
Map of the Gobi Desert in Mongolia. The map shows the six areas named GGA 04-07-08-10-11-12, where bats were captured.

The University of Florence (UniFI), in collaboration with the National University of Mongolia, the University of Verona, the Italian Institute for Environmental Protection and Research (ISPRA), and the Istituto Zooprofilattico Sperimentale della Lombardia e dell’Emilia Romagna (IZSLER), organized a 25-day expedition in the Gobi Desert. Multidisciplinary scientists explored six oases (Figure 1) as follows:

- GGA 04 (LAT 44,92143499; LONG 96,66617872): 1 bat captured;
- GGA 07 (LAT 43,35308333; LONG 96,34411667): 20 bats captured;
- GGA 08 (LAT 43,30285000; LONG 97,77906667): 8 bats captured;
- GGA 10 (LAT 42,88606667; LONG 98,89566667): 15 bats captured;
- GGA 11 (LAT 42,88171667; LONG 98,81793333): 5 bats captured;
- GGA 12 (LAT 43,24652002; LONG 99,00125125): 25 bats captured.

In Oases GGA 08 and GGA 12, two distinct points, A and B, were identified within the same area, specifically GGA 08A and GGA 08B and GGA 12A and GGA 12B, respectively.

A total of 74 bats were captured using mist nets; each bat was identified based on its morphological characteristics directly on site. Before their release, a portion of the patagium and fecal samples were collected from each bat. All samples were stored in appropriate eNAT Guanidine medium (eNAT^®^ Molecular Collection and Preservation Medium, Copan Italia S.p.A., Brescia, Italy) at room temperature until they arrived at the laboratory.

All procedures involving the capture, handling, and sampling of bats were carried out in full compliance with international ethical guidelines for the use of wild mammals in research and according to the legal requirements of the countries involved. This study was conducted under the supervision and approval of the relevant Mongolian authorities and institutional committees. Bats were handled by trained personnel, and all efforts were made to minimize stress and ensure animal welfare during field procedures.

### 2.2. Ecological Context and Sampling Conditions

From a phytogeographical perspective, the characteristics of the oases have been described in detail in Esposito et al., 2024 [16]. To maximize bat catchability, sampling was carried out in May, at the end of the dry season, when midday temperatures ranged between 20 °C and 27 °C. During this period, the scarcity of water compels bats to gather at water sources, increasing the chances of capture. Each oasis was surveyed for two consecutive days. Due to this limited sampling window, we did not collect data on bat density or species diversity.

However, we recorded notable habitat features related to water availability, which may influence bat aggregation and thus potentially affect viral transmission dynamics. In GGA 04 and GGA 12, there were two small circular lakes, each approximately 30 m in diameter. GGA 07 had a small pond with limited water availability. In GGA 08, we found a small river approximately 200 m in length. GGA 10 and GGA 11 each had a small pond. These water bodies likely act as focal points for bat activity during the dry season, facilitating contact among individuals and possibly enhancing opportunities for interspecies viral transmission.

### 2.3. Molecular Analysis

Total nucleic acid (RNA and DNA) was simultaneously extracted from 74 bat fecal samples, and their patagia in eNAT medium (Copan Diagnostics, Brescia, Italy) was extracted using the QIAamp DSP Virus/Pathogen Mini Kit (Cat. No. 61904, Qiagen, Hilden, Germany) on a Qiasymphony^®^ SP automated extraction system (Qiagen, Hilden, Germany) according to the manufacturer’s instructions.

According to the European bat identification key, all bat species were identified taxonomically based on both morphological characteristics [17] and a molecular method performed on the patagium [18].

Nucleic acids extracted from all fecal samples were screened using a panel of molecular assays targeting viruses from a broad range of taxonomic groups, including coronaviruses (CoVs), Mammalian orthoreoviruses (MRVs), Influenza A virus (AIV), pestiviruses, paramyxoviruses, herpesviruses, adenoviruses (ADVs), orthoflaviviruses, and phleboviruses. The various PCR protocols employed for detecting these viral agents are detailed in Table 1. The primer and probe sequences used for virus detection are reported in Supplementary Table S1.

A specific RT-PCR assay was developed to identify the Influenza A virus in bats by amplifying a 402 bp region of the PB1 gene. RNA was amplified using the modified method described by Kandeil et al. [22]. Briefly, we employed the QuantiTect^®^ Probe RT-PCR Kit (Cat. No. 204443, Qiagen, Hilden, Germany) under the following conditions: 50 °C for 30 min, 95 °C for 15 min, followed by 40 cycles at 94 °C for 1 min, 50 °C for 1 min, and 72 °C for 1 min, concluding with 72 °C for 10 min.

To detect herpesvirus, we employed the PanHerpesvirus Nested PCR method described by VanDevanter et al., 1996 [27], targeting a region between 190 and 250 bp in the DNA polymerase gene. Nested PCR was performed using the HotStartTaq^®^ Master Mix Kit (Cat. No. 203445, Qiagen, Hilden, Germany). Both PCRs were conducted under identical conditions: 95 °C for 15 min, followed by 45 cycles at 94 °C for 30 s, 46 °C for 30 s, 72 °C for 1 min, and finally, 72 °C for 10 min.

The modified protocol described by Diakoudi et al. (2019) [28] was adopted to identify adenoviruses. It consists of a nested PCR that amplifies a region between 318 and 324 bp of the DNA polymerase gene using the Platinum^®^ Taq DNA Polymerase Kit (Cat. No. 10966034, Invitrogen, Carlsbad, CA, USA). The conditions for the first PCR cycle were 94 °C for 2 min, followed by 40 cycles at 94 °C for 30 s, 55 °C for 30 s, and 72 °C for 1 min, concluding with 72 °C for 10 min. The same conditions were established for the second PCR, with annealing performed at 48 °C for 30 s (Table 1). The remaining viral agents were treated using the PCR methods outlined in the authors’ protocols (Table 1).

### 2.4. Sanger Sequencing

The PCR-positive products were sequenced using the Sanger method; briefly, the amplified products were purified using ExoSAP-IT™ Express PCR Product Cleanup Reagent (Cat. No. 75001, ThermoFisher Scientific, Waltham, MA, USA) and FastAP™ Thermosensitive Alkaline Phosphatase (Cat. No. EF0651, ThermoFisher Scientific, Waltham, MA, USA) in accordance with the manufacturer’s instructions. Sanger sequencing was performed with the BigDye^®^ Terminator v1.1 Cycle Sequencing Kit (Cat. No. 4337450, Applied Biosystems, Foster City, CA, USA) employing the same primers used for PCR amplification. Sequencing reactions were subsequently purified using the BigDye^®^ XTerminator™ Purification Kit (Cat. No. 4376486, Applied Biosystems, Waltham, MA, USA) and separated on a 3500 xl Genetic Analyzer (Applied Biosystems, ThermoFisher Scientific, Waltham, MA, USA) following the manufacturer’s instructions.

### 2.5. Statistical Analysis

To investigate the association between virus positivity and categorical variables (species, sex, and provenance), univariate analyses were performed. Initially, contingency tables were constructed to summarize the number of positive and negative individuals for each category within these variables. Given the presence of small sample sizes and low expected cell counts (<5) in several contingency tables, the conventional chi-square test of independence was deemed inappropriate due to its reliance on asymptotic approximations. Therefore, Fisher’s Exact Test, which calculates exact *p*-values irrespective of sample size, was applied to test the null hypothesis of no association between infection status and each categorical variable. Moreover, *p*-values < 0.5 were considered significant. Prevalence (%) of positive individuals was calculated for each species within each geographic location.

## 3. Results

In May 2022, 74 fecal samples were collected from insectivorous bats captured during an expedition in the Gobi Desert in Mongolia.

**Figure 2:**
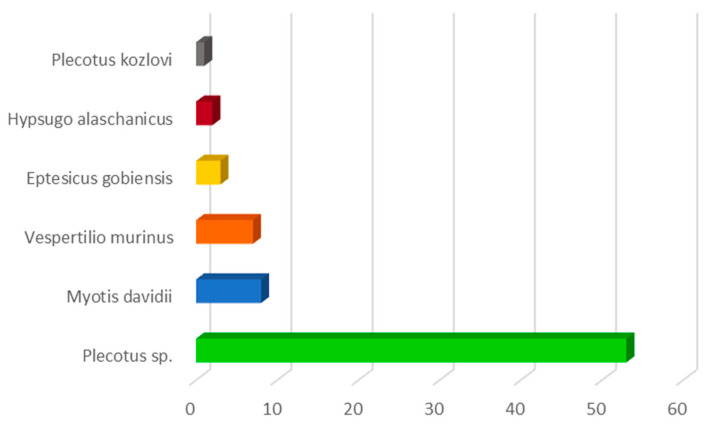
Number of individuals of bat species captured in the Gobi Desert, Mongolia.

Morphological identification revealed that the most representative genus found in the Gobi Desert was *Plecotus*, followed by individuals of the *Hypsugo*, *Vespertilio*, and *Myotis* species. Subsequent molecular analysis of patagium portions facilitated a more precise identification of the various bat species, confirming a notable presence (73%) of bats belonging to *Plecotus* spp. with 54 individuals, including one *Plecotus kozlovi*. These were followed by eight *Myotis davidii* (10.8%), seven *Vespertilio murinus* (9.5%), three *Eptesicus gobiensis* (4%), and two *Hypsugo alaschanicus* (2.7%) (Figure 2).

Eleven samples (14.86%), ten from *Plecotus* spp. and one from the *Myotis davidii* species, tested positive for coronavirus at the nested PCR (Table 2). Four bats were captured in the GGA10 area, five in GGA12B, and two in GGA12A (Figure 1).

The eleven CoV-positive samples, analyzed by BLAST (https://blast.ncbi.nlm.nih.gov/Blast.cgi, accessed on 10 July 2025) showed nucleotide identity ranging from 94.94% to 95.65% with the MERS-related CoV strain BatCoV_B20-180, which was detected in *Rhinolophus ferrumequinum* in South Korea in 2020 (GenBank: ON378807.1), as shown in Table 2. Phylogenetic reconstruction using the maximum likelihood method based on the partial RdRp gene confirmed the clustering of the detected sequences within the Merbecovirus subgenus and their close evolutionary proximity to BatCoV_B20-180, isolated from *Rhinolophus ferrumequinum* in South Korea (Supplementary Figure S1).

Two (2.70%) bats, one *Vespertilio murinus* and one *Plecotus* sp., collected from the GGA07 area, tested positive for MRV. BLAST analysis confirmed the positivity for MRV, demonstrating a high degree of sequence identity with the MRV strain WIV4 detected in *Hipposideros* sp. in China in 2011 [29] (Gene Bank: KT444532.1) and with the MRV isolate SI-MRV06 segment L1 lambda 3 (L1) gene/MRV 3 strain3/*Rhinolophus_hipposideros*/Italy/191797/2011 lambda 3 protein (L1) gene identified in the same year in Italy [30] (Gene Bank: JX028411.1) (Table 3).

Three samples (3.3%) from *Plecotus* sp. and *Myotis davidii* in GGA10-11 and 12 tested positive for herpesvirus (Figure 1). BLAST analysis of one sequence (PV126593) belonging to the Myotis genus resulted in a positive identification for herpesvirus, showing an 82.11% similarity with the Bat herpesvirus YBS33_O gene for DNA polymerase identified in 2022 in Japan [31] (Gene Bank: LC810607). In contrast, the two sequences from Plecotus, which are identical, demonstrated a low percentage of similarity with hedgehog and bat sequences (Table 4) [32]. However, further analyses based on the sequencing of a larger portion of the genome must be performed to confirm the results obtained.

The samples examined resulted negative for Influenza A virus, pestivirus, flavivirus, phlebovirus, paramyxovirus, or adenovirus.

Considering the low positivity rates for Mammalian orthoreovirus and herpesvirus, statistical analysis was conducted only for the results related to coronavirus testing. The univariate analysis revealed that coronavirus positivity was predominantly detected in the species *Plecotus*, with prevalence rates of 36.4% (4/11) and 26.1% (6/23) in the GGA10 and GGA12 areas, respectively. In *Myotis* from GGA12, one out of two individuals tested positive (50%), but the low sample size limits reliable interpretation. All other species and locations showed zero prevalence. Fisher’s Exact Test confirmed a statistically significant association between provenance and coronavirus positivity (*p* = 0.03). Sex and species alone were not significantly associated with infection status in univariate tests. Table 5 shows details of sample numbers and the proportion of positive bats for each oasis.

## 4. Discussion

The intrinsic characteristics of bats, including the large number of species, their status as the only mammals capable of flight, long-distance migration, gregarious behavior, specialized immune systems, and diversity of habitats, render them particularly fascinating animals for the study of their potential roles as reservoirs and vectors of pathogens, primarily viruses. Consequently, they may play an important role in the etiopathogenesis of zoonotic and emerging diseases.

Although the phenomenon of coevolution between individuals of this species and some viral species (particularly Lyssaviruses) is well known and studied, in recent decades, the possibility that certain viral agents, whether directly or indirectly, have passed from bats to other wild and domestic animal species, as well as humans, has become increasingly strong. In particular, the emergence of Henipavirus diseases (Nipah virus in pigs and Hendra virus in equines), both of a zoonotic nature, and the more recent pandemic of SARS-CoV-2, whose origin, although not yet fully clarified, is linked to the evolution of viral ancestors present in some bat species, have significantly contributed to reinforcing and emphasizing the fundamental role that bats play in the so-called spillover phenomenon.

Moreover, some of our recent studies have shown that the presence of rarely reported viral agents with an uncertain pathogenic role, although potentially zoonotic, is anything but uncommon. This is the case, for example, for the Vaprio ledantevirus [33], the Hypsugopox virus [34], and the Issyk-kul virus [35], all of which have been reported in the Po Valley over the last decade, along with many other viruses, including orthoreovirus, coronavirus, adenovirus, paramyxovirus, and so on.

Overall, the results obtained indicate a viral presence that aligns with findings from investigations carried out in Italy, as well as across Europe and other regions of the world [36]. There is a greater prevalence of MERS-like coronavirus strains, along with a sporadic presence of reoviruses and herpesviruses. The analysis indicates that coronavirus infection in bats appears to be spatially clustered and species-specific, with *Plecotus* bats in certain geographic areas exhibiting higher prevalence. The detection of positive individuals primarily in GGA10 and GGA12 suggests local factors may influence viral circulation. However, the small sample sizes in several species–area combinations limit the generalizability of these findings. In this regard, although detailed ecological data on bat density and species diversity were not collected, the habitat characteristics of the oases provide useful context. Sampling was conducted in May, at the end of the dry season, when water scarcity likely increased bat aggregation around permanent or semi-permanent water sources. Specifically, several oases, such as GGA 04 and GGA 12, contained small lakes, while GGA 08 featured a short river, and others contained ponds. These water bodies may have acted as focal points for interspecies contact and, consequently, viral transmission. The spatial clustering of positive cases in water-rich oases like GGA 10 and GGA 12 may reflect this ecological influence, although further data are required to confirm such associations.

The findings from the Gobi Desert study, particularly the detection of MERS-related coronaviruses in *Plecotus* spp. and *Myotis davidii*, are consistent with results from northwestern China and Central Asia, where related coronaviruses have been detected in ecologically similar bat species such as *Rhinolophus* and *Myotis* [37,38]. The identification of Mammalian orthoreoviruses in *Vespertilio murinus* and *Plecotus* sp. aligns with previous reports from China, where similar strains were found in *Hipposideros* and *Miniopterus* bats [29], and from Europe, suggesting a widespread Eurasian distribution. Likewise, the detection of herpesviruses in *Plecotus* and *Myotis* aligns with findings from Japan, where genetically diverse Bat herpesviruses have been characterized [39]. The absence of flaviviruses, phleboviruses, and influenza viruses in the Gobi samples reflects similar trends observed in arid or sparsely populated areas, where environmental conditions and lack of competent vectors may hinder their circulation [40]. Overall, coronaviruses emerge as the most frequently detected viruses in bats from arid regions, while the distribution of other viral families appears to be more sporadic and ecologically driven.

Despite the valuable insights generated by this study, several methodological limitations must be considered. First, the relatively small sample size, both overall and within individual bat species or geographic areas, may have reduced the statistical power to detect meaningful associations or less prevalent viral infections. Moreover, the molecular assays employed may show variable sensitivity in detecting low-viral-load or genetically divergent viral strains, possibly leading to false negatives. This limitation is particularly relevant when working with field samples, which may have been subjected to suboptimal storage or transport conditions. However, it is important to note that all PCR products obtained in this study were confirmed by Sanger sequencing, which provides a high level of specificity and serves as a strong validation of the amplified targets. This approach effectively minimizes the risk of false positives and negatives, strengthening the reliability of the molecular findings reported. Furthermore, the investigation provides only a temporal snapshot of viral shedding, without accounting for seasonal or longitudinal trends. To better understand virus–host dynamics and the ecology of viral maintenance in bat populations from the Gobi Desert, future research should incorporate longitudinal sampling and serological surveys. These would allow for detection of past exposures not captured by molecular methods and help to identify patterns of persistence and potential spillover.

The failure to identify adenoviruses [28] and paramyxoviruses [24,41], which are relatively common in the samples analyzed in Northern Italy, is somewhat surprising. Meanwhile, the absence of other viral agents such as flaviviruses, phleboviruses, pestiviruses, and influenza viruses reinforces previous data. However, apart from the *Plecotus* species, in most cases the samples are too small to truly rule out the infections investigated.

Consequently, on one hand, the presence of agents from certain viral species has been established, indicating a likely co-evolution between the virus and its host, as well as a form of tolerance towards these agents that aids their spread and transmission within the same group of bats and potentially to other species. This supports the notion of their role as reservoirs and vectors, even over long distances through migration. On the other hand, it is plausible that bats play a considerably smaller role in the transmission of other viral families, with mechanisms being mediated by different vectors such as mosquitoes, ticks, and sandflies (flaviviruses and phleboviruses), as well as sedentary and migratory birds (flaviviruses and influenza viruses).

Despite their role as reservoirs, the low interaction between humans and bats, similar to what presumably occurs in the Gobi Desert, could indicate that interspecies transmission should be considered very sporadic. However, drastic changes in natural ecosystems due to deforestation, industrialization, and human activities could increase and favor direct or indirect contact between humans and the natural reservoirs.

Consequently, we cannot rule out the possibility of future spillover. Implementing surveillance strategies and early detection methods for new and emerging agents is essential for curtailing the emergence and spread of viral infections to new hosts and regions.

## 5. Conclusions

The availability of consolidated procedures and a panel of validated molecular diagnostic tests, which can be implemented to include additional viral groups whose presence has been described in bats (e.g., astrovirus, parvovirus, poxvirus, morbillivirus, etc.), is a necessary prerequisite for conducting virological screening that can be used in various geographical settings for research purposes and a more reliable evaluation of the risks arising from the interaction between bats and other animal species, including humans.

Although our study was limited by small sample sizes and the lack of data on bat density and diversity, the ecological features of the sampled oases, particularly the presence of water bodies during the dry season, likely influenced bat aggregation and viral transmission dynamics. These findings highlight the importance of considering environmental and seasonal factors in future surveillance efforts.

Finally, enhancing the overall knowledge of areas that are still partially free from damage caused by human activities could help to inform actions to mitigate impacts on natural environments. However, the potential for future zoonotic spillover events cannot be excluded. Continuous ecological and virological monitoring, combined with early detection systems, remains a cornerstone of global preparedness against emerging infectious diseases.

## Figures and Tables

**Table 1:** Summary of PCR/RT-PCR protocols used in this study. This table summarizes the main characteristics of the PCR and RT-PCR protocols used for virus detection, grouped by RNA and DNA viruses. RNA viruses (**A**); DNA viruses (**B**).

(A)				
Virus	Assay Type	Target Gene	Amplicon Size (bp)	Reference
Coronavirus	Nested RT-PCR	RdRp	440	[19]
Mammalian Orthoreovirus	Real-time RT-PCR/Nested RT-PCR	RdRp/L1	344	[20,21]
Influenza A Virus	RT-PCR	PB1	402	[22]
Pestivirus	Real-time RT-PCR	5′ NTR	—	[23]
Paramyxovirus	Nested RT-PCR	L protein	200–500	[24]
Flavivirus	RT-PCR	NS5	240	[25]
Phlebovirus	RT-PCR	S segment	370	[26]
(**B**)				
**Virus**	**Assay Type**	**Target Gene**	**Amplicon Size (bp)**	**Reference**
Herpesvirus	Nested PCR	DNA polymerase	190–250	[27]
Adenovirus	Nested PCR	DNA polymerase	318–324	[28]

**Table 2:** Coronavirus-positive samples.

ID Sample	Bat Species	Sex	Location	Virus Detected	Sequence Target	Target	Gene Bank	Nucleotide Identity
283108/33	*Plecotus* sp.	M	GGA10	CoV	RdRp	440 bp	PV126580	94.94–95.65% with MERS-related CoV BatCoV_B20-181 (ON378807)
283108/36	*Plecotus* sp.	M	GGA10				PV126581	
283108/41	*Plecotus* sp.	M	GGA10				PV126582	
283108/44	*Plecotus* sp.	M	GGA10				PV126583	
283108/51	*Plecotus* sp.	F	GGA12B				PV126584	
283108/55	*Plecotus* sp.	F	GGA12B				PV126585	
283108/56	*Plecotus* sp.	M	GGA12B				PV126586	
283108/57	*Plecotus* sp.	F	GGA12B				PV126587	
283108/62	*Plecotus* sp.	M	GGA12B				PV126588	
283108/63	*Myotis davidii*	F	GGA12A				PV126589	
283108/69	*Plecotus* sp.	F	GGA12A				PV126590	

**Table 3:** Mammalian orthoreovirus-positive samples.

ID Sample	Bat Species	Sex	Location	Virus Detected	Sequence Target	Target	Gene Bank	Nucleotide Similarity
283108/14	*Vespertilio murinus*	M	GGA07	MRV	λ3 RdRp	344 bp	PV126592	100% with MRV isolate WIV4 lambda-3 gene
283108/9	*Plecotus* sp.	M	GGA07				PV126591	98.81% with MRV isolate SI-MRV06 segment L1 lambda 3 (L1) gene/MRV 3 strain T3/Rhinolophus_hipposideros/Italy/191797/2011 lambda 3 protein (L1) gene

**Table 4:** Herpesvirus-positive samples.

ID Sample	Bat Species	Sex	Location	Virus Detected	Sequence TARGET	Target	Gene Bank	Nucleotide Similarity
283108/45	*Myotis davidii*	M	GGA10	Herpes virus	DNA Polymerase gene	190–250 bp	PV126593	82.11% Bat herpesvirus YBS33_Myotis macrodactylus_Japan_2022_(LC810607)
283108/47	*Plecotus* sp.	M	GGA11				/	80% hedgehog herpesvirus isolate XT918-16_UK_2016 (MG253639) 76.64% Bat herpesvirus isolate HP/11HN110_China_2011 (KR261845)
283108/62	*Plecotus* sp.	M	GGA12B				/	

**Table 5:** Summary of the proportion of bats testing positive for coronavirus, with the number of positive bats/total samples collected shown in brackets.

Oasis	*Epseticus*	*Hypsugo*	*Myotis*	*Plecotus*	*Vespertilio*
GGA04	-	-	0% (*n* = 0/1)	-	-
GGA07	-	0% (*n* = 0/1)	0% (*n* = 0/1)	0% (*n* = 0/15)	0% (*n* = 0/3)
GGA08	0% (*n* = 0/2)	0% (*n* = 0/1)	0% (*n* = 0/1)	0% (*n* = 0/2)	0% (*n* = 0/2)
GGA10	0% (*n* = 0/1)	-	0% (*n* = 0/3)	36.4% (*n* = 4/11)	-
GGA11	-	-	-	0% (*n* = 0/3)	0% (*n* = 0/2)
GGA12	-	-	50% (*n* = 1/2)	26.1% (6/23)	-

## Data Availability

Data are contained within this article.

## Supplementary Materials

The following supporting information can be downloaded at: https://www.mdpi.com/article/10.3390/pathogens14090870/s1. Table S1: Primer and probe sequences used for virus detection; Figure S1: Maximum likelihood (ML) phylogenetic tree based on the partial RdRp gene sequences of bat coronaviruses and reference coronavirus sequences. Sequences derived from bats in this study are highlighted in green. The ML tree was inferred using the IQ-TREE web server with 1000 bootstrap replicates. Bootstrap values ≥ 70 are indicated in the corresponding nodes.
