# Supplementary material for: Exploring Bat–Virus Interactions: Insights from a Study in the Gobi Desert
Source: Pathogens. 2025 Sep 2;14(9):870. doi: 10.3390/pathogens14090870 (PMC12472993; doi:10.3390/pathogens14090870)
Supplement: Supplementary file 1 [file pathogens-14-00870-s001.zip › pathogens-3781504 Table S1.pdf]

Supplementary Table S1. Primer and probe sequences used for virus detection

Virus	Assay Type	Target Gene	Primer/Probe Name	Sequence (5'–3')	Amplicon Size (bp)	Reference
**Coronavirus**	Nested RT-PCR	RdRp	PANCOVchu-1F	GGKTGGGAYTAYCCKAARTG	440	[18]
			PANCOVchu-2R	TGYTGTSWRCARAAYTCRTG		
			PANCOVchu-3F	GGTTGGGACTATCCTAAGTGTGA		
			PANCOVchu-4R	CCATCATCAGATAGAATCATCAT		
**Mammalian Orthoreovirus**	Real-time RT-PCR	RdRp	BATREO-F	CACCATGTCAAGCTGCTCCC	344	[19]
			BATREO-R	ACCGCCATGTATGTCCTCCAG		
			BATREO-PROBE	fam-CCCAGTCGCGGTCATTACCACTCCG-BBQ		
	Nested RT-PCR	L1	L1-RV5-F	GCATCCATTGTAAATGACGAGTCTG		[20]
			L1-RV6-R	CTTGAGATTAGCTCTAGCATCTTCTG		
			L1-RV7-F	GCTAGGCCGATATCGGGAATGCAG		
			L1-RV8-R	GTCTCACTATTCACCTTACCAGCAG		
**Influenza A Virus**	RT-PCR	PB1	FLUAPB1-F	ATGATGATGGGNATGTTYAAYATG	402	[21]
			FLUAPB1-R	CNGGNCCNAKDTCRYTRTTDATCAT		
**Pestivirus**	Real-time RT-PCR	5′ NTR	BVD-190-F	GRAGTCGTCARTGGTTCGAC	—	[22]
			BVD-V326-R	TCAACTCCATGTGCCATGTAC		
			BVD-TQ-PROBE	fam-TGCYAYGTGGACGAGGGCATGC-tamra		
**Paramyxovirus**	Nested RT-PCR	L-protein	PAR-F1	GAAGGITATTGTCAIAARNTNTGGAC	200–500	[23]
			PAR-F2	GTTGCTTCAATGGTTCARGGNGAYAA		
			PAR-R	GCTGAAGTTACIGGITCICCDATRTTNC		
**Flavivirus**	RT-PCR	NS5	MAMD-F	AACATGATGGGRAARAGRGARAA	240	[26]
			cDF2-REV	GTGTCCCAGCCGGCGGTGTCATCAGC		
**Phlebovirus**	RT-PCR	S segment	PHLEBO F1	TTTGCTTATCAAGGATTTGATGC	370	[27]
			PHLEBO F2	TTTGCTTATCAAGGATTTGACC		
**Herpesvirus**	Nested PCR	DNA polymerase	DFA For	GAYTTYGCNAGYYTNTAYCC	190–250	[24]
			ILK For	TCCTGGACAAGCAGCARNYSGCNMTNAA		
			KG1 Rev	GTCTTGCTCACCAGNTCNACNCCYTT		
			TGV For	TGTAACTCGGTGTAYGGNTTYACNGGNGT		
			IYG Rev	CACAGAGTCCGTRTCNCCRTADAT		
**Adenovirus**	Nested PCR	DNA polymerase	PolFouter For	TNMGNGGNGGNMGNTGYTAYCC	318–324	[25]
			PolRouter Rev	GTDGCRAANSHNCCRTABARNGMRTT		
			PolFinner For	GTNTWYGAYATHTGYGGHATGTAYGC		
			PolRonner Rev	CCANCCBCDRTTRTGNARNGTRA		
