# Supplementary figures and images for: Exploring Bat–Virus Interactions: Insights from a Study in the Gobi Desert
Source: Pathogens. 2025 Sep 2;14(9):870. doi: 10.3390/pathogens14090870 (PMC12472993; doi:10.3390/pathogens14090870)

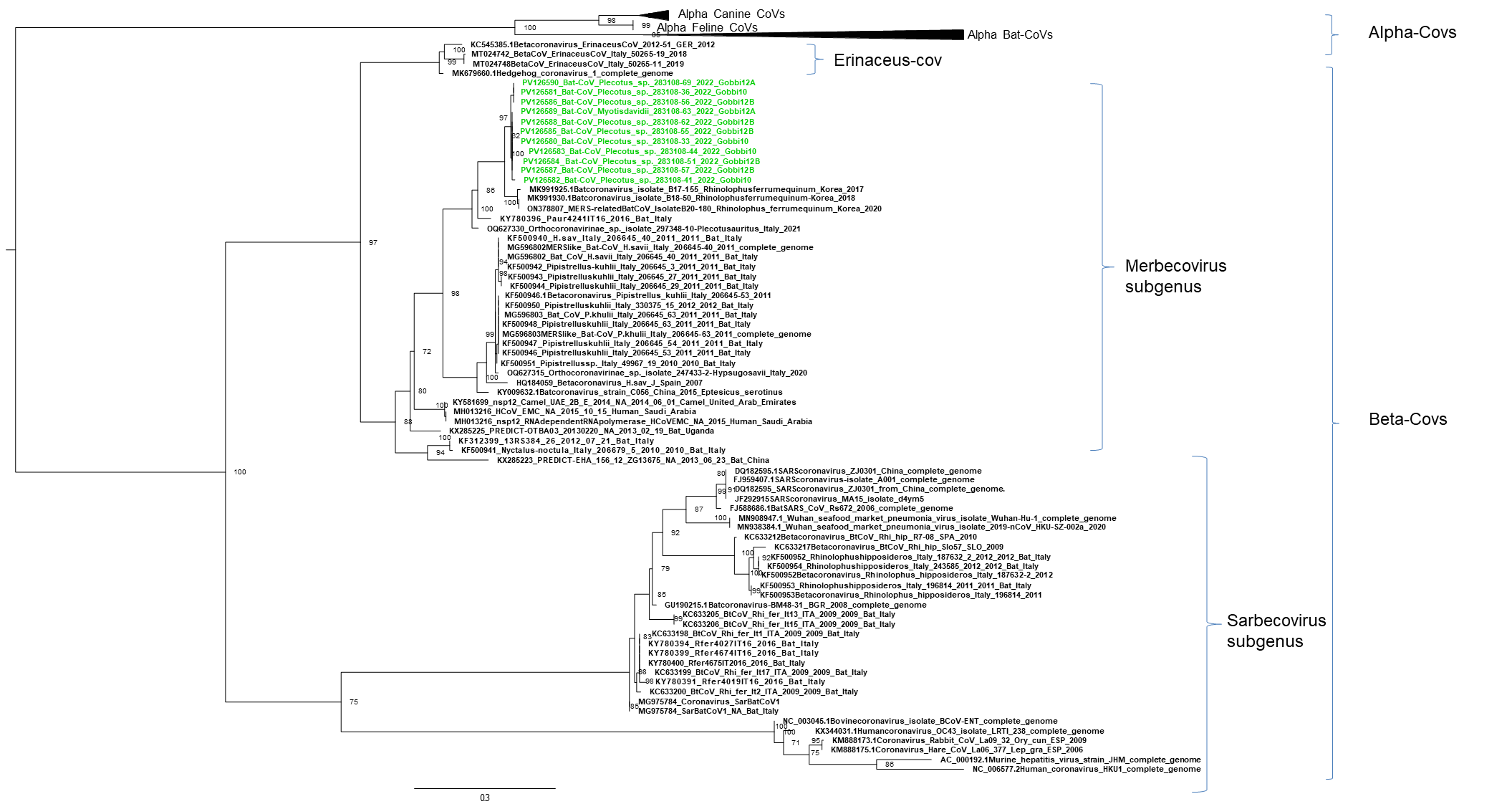

Supplement: Supplementary file 1 [file pathogens-14-00870-s001.zip › pathogens-3781504 Figure S1.tif]
